# Native Plants Can Strengthen Urban Green Infrastructure: An Experimental Case Study in the Mediterranean-Type Region of Central Chile

**DOI:** 10.3390/plants14193025

**Published:** 2025-09-30

**Authors:** Javier A. Figueroa, Rosa Chandía-Jaure, Andrés Cataldo-Cunich, Sergio Cárdenas Muñoz, Francisca Fernández Cano

**Affiliations:** 1 Centro de Investigación Arquitectónica, Urbanística y del Paisaje, Facultad de Ingeniería y Arquitectura, Universidad Central de Chile, Avenida Santa Isabel 1186, Santiago 8330601, Chile; 2 Departamento de Planificación y Ordenamiento Territorial, Universidad Tecnológica Metropolitana, Dieciocho 161, Santiago 8330378, Chile; rchandia@utem.cl (R.C.-J.); andres.cataldo@utem.cl (A.C.-C.); 3 Facultad de Ingeniería y Arquitectura, Universidad Central de Chile, Avenida Santa Isabel 1186, Santiago 8330601, Chile; scardenasm@ucentral.cl; 4 Carrera de Arquitectura del Paisaje, Facultad de Ingeniería y Arquitectura, Universidad Central de Chile, Avenida Santa Isabel 1186, Santiago 8330601, Chile; ffernandezc@ucentral.cl

**Keywords:** urban plant, native plant, plant survival, plant growth, water use, central Chile

## Abstract

In Santiago, Chile, urban plants are highly vulnerable to drought or climate change. We hypothesize that would find high growth and survival rates in conditions of water scarcity among native species of central Chile. The goal was to determine the effect of the year season and an irrigation gradient on the survival and growth of native plant, in order to evaluate potential plant for use in urban green areas of central Chile. Four plots of 20 m^2^ were located in the Santiago center. In June 2024 twelve species were planted and from November 2024 to March 2025 were irrigated with 13.3, 10.1, 1.7 and 1.4 L/m^2^/day. The GLM and Kaplan–Meier survival analyses were used. Shoot growth rate was highly variable among species, among irrigation treatments applied, and among year seasons. Eight species showed water-related growth and shoot growth during the winter was very small and higher in spring. Two species showed evidence of water-related survival; in the other 10 species, no significant differences were found between irrigation treatments. Winter was the season with the highest survival rates for eleven species. In conclusion, the results suggest that native plants can achieve high survival rates with limited irrigation. This highlights their potential for use in the urban area in Mediterranean-type climates where rainfall is expected to be low due to climate change.

## 1. Introduction

In Santiago, Chile, urban plants are highly vulnerable to drought, as they are mainly exotic from temperate origins. However, experimental studies seeking evidence of potential native species in central Chile that could replace and develop the current urban green infrastructure are limited in number.

Urban vegetation is known to improve air quality [1], reduce summer heat island temperatures, lower cooling costs [2], reduce storm-water runoff [3,4], reduce atmospheric carbon dioxide [5], contribute to the conservation of biodiversity [6], and improve various social and individual development indicators among residents [7,8,9,10,11]. The average opinion of urban populations on urban vegetation is very positive, with particular value placed on shade, aesthetics, air quality and noise reduction [12].

The lack of vegetation in central Chilean cities (for example, 5.7% of Santiago’s population has access to >9 m^2^ green space/inhabitant) is accentuated by the unsustainable design of urban areas [13]. This is characterized by high maintenance and irrigation requirements and is driven by the selection of plant species that are not adapted to the urban conditions of the region (e.g., soil, atmosphere, water and nutrients). Urban vegetation in cities in central Chile is also deteriorated and stressed by climate change, along with adverse growing conditions including compacted soil, extreme heat, lack of nutrients, drought, damage from cars, pruning, and vandalism [14]. Therefore, any plan for the development or replacement of urban vegetation in the region should thus be based on experimental evidence in real urban areas, at least considering the survival and growth of potential urban plants. Unfortunately, experimental studies with native species are not common in urban areas located in the Mediterranean-type climate region of central Chile.

Vegetation survival and growth in an urban setting depends on planting location, installation, and post-planting care, between others [15,16,17,18,19]. For example, the most common environmental conditions influencing urban tree mortality are related to water stress, nutrient deficiency, and soil compaction [20,21]. Plant species that successfully colonize and persist in public areas would likely possess traits for stress-tolerance or avoidance. In seasonally dry ecosystems, such as those of central Chile, water is the limiting factor for survival and vegetation growth [22]. In order to design urban areas that support urban biodiversity for future generations, it is important to understand which species persist and how plant communities change over time.

Because urban tree care can be inconsistent during and after planting, it is important to choose plant species that are likely to survive and grow well with minimal additional care [23]. Published studies have primarily focused on the survival and growth of urban trees, and most research on urban tree success originates from experiments conducted in relatively controlled nursery settings rather than in environments exposed to urban conditions. However, urban herbs and shrubs can contribute to the diversity and functionality of urban ecosystems. Their survival and growth are influenced by the urban habitat.

A study of this characteristic has important implications for urban species selection in a changing climate. Cities located in Mediterranean-type climates are particularly vulnerable to climate change [24]. Mainly are predicted to face reduced annual precipitation, temperature increase, and intensified rainfall events [25]. In Santiago, Chile, urban trees are highly vulnerable to drought because they are mainly exotic species that are not adapted to water scarcity [26]. This forces municipalities to use potable water for irrigation [24]. Assessing the tolerance or resistance of potential urban plants to climate change helps architects and urban landscape designers select plants suited to cities facing water scarcity [27].

In this study, we estimated the survival and growth rates of seedlings over 10 consecutive months through seasonal monitoring of 12 potential native species of central Chile for use in urban green infrastructure in Santiago. The analyses used the cumulative survival rate and growth index of the shoots per season for each species tested. These indicators are widely used in demography and population ecology.

Although species generally differ in their response to water restriction, we hypothesize that would find high survival rates among native species in central Chile during the first year of the trial in an urban area. The main objective of the study was to determine the effect of the year season and an irrigation gradient on the survival and growth of native species in order to evaluate potential plant for use in urban green infrastructure in Mediterranean-type climate cities in central Chile.

## 2. Results

### 2.1. Shoot Growth

**Figure 1:**
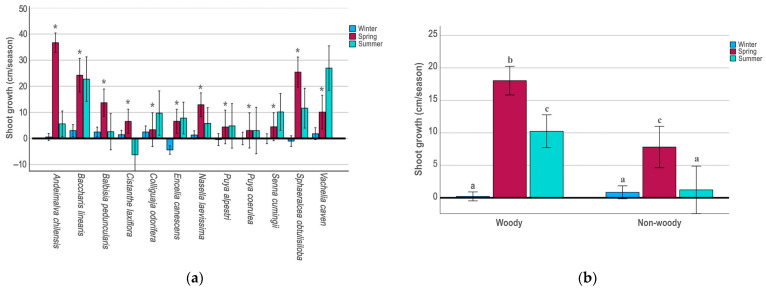
(**a**) Shoot yield index for winter, spring, and summer seasons in 12 native plant species from central Chile. Bars represent ±1 E.D. * indicate statistically significant differences among seasonal for each species assessed according to Bonferroni test (*p* ≤ 0.05); (**b**) shoot yield index for winter, spring, and summer seasons for woody and non-woody species combined. Bars represent ±1 E.D. Different letters (a–c) above the bars indicate statistically significant differences among year seasonal according to Bonferroni test (*p* ≤ 0.05).

Shoot growth varied among species (Figure 1a). The species with the highest average growth during all the study periods were the shrubs *Baccharis linearis* (16.7 ± 1.8 cm), *Andeimalva chilensis* (14.3 ± 1.1 cm), *Sphaeralcea obtusiloba* (12.0 ± 1.6 cm), and the tree *Vachelia caven* (13.0 ± 1.8 cm). At the opposite end of the spectrum, the woody *Cistanthe laxiflora* (0.6 ± 1.3) and the succulent herbs *Puya alpestri* (2.9 ± 1.8 cm) and *P. coerulea* (2.0 ± 1.9 cm) showed the lowest average growth of the species studied during the study period. The average growth of woody species (9.5 ± 0.6 cm) during the study period was higher than that of non-woody species (3.3 ± 0.8 cm), with a significant difference between both (F = 37.98; *p* < 0.001).

Eight species showed evidence of water-sensitive growth (Table 1). In all this species, individuals subjected to a higher irrigation volume tended to show greater shoot average growth. Overall, the combined water-sensitive growth species in the treatment with higher irrigation (average 9.9 cm throughout the test) was >50% higher than the combined water-sensitive growth species in the treatment with lower irrigation (average 3.3 cm throughout the test).

In particular, the woody *Cistanthe laxiflora* (−3.2 ± 1.4 cm), the herb *Nasella laevissima* (0.9 ± 1.2 cm), the succulents *P. alpestri* (1.3 ± 1.1 cm) and *P. coerulea* (0.02 ± 1.1 cm) showed very low average growth in the lowest irrigation volume treatment (Table 1). In fact, *C. laxiflora* and *S. cumingii* stand out because they were the species that showed negative growth in response to the lowest water treatments of 1.4 L/m^2^/day and 1.7 L/m^2^/day, respectively (Table 1).

In the other 4 tested, *Baccharis linearis*, *Balbisia peduncularis*, *Encelia canescens*, and *Sphaeralcea obtusiloba*, did not exhibit water-sensitive growth (Table 1).

In a seasonal analysis, shoot growth was highly variable among year seasons in all the species tested (Table 2). In the 12 species, shoot growth during the winter season was very small, it was even negative in *E. canescens* (F = 12.25; *p* ≤ 0.001), and in *S. obtusiloba* (F = 19.65; *p* ≤ 0.001) although without differences among treatments in both species (Table 2). However, spring was the most productive season, except for the tree *V. caven* (Figure 1a), which grew faster in summer (F = 20.31; *p* < 0.001). In contrast, growth in *C. laxiflora* decreased significantly and was negative during the summer (F = 10.09; *p* < 0.01).

Globally, the combined growth of the 12 species was also significantly higher in spring than in winter or summer (F = 52.53; *p* < 0.001). A similar pattern, although more pronounced, was found when comparing woody species with non-woody species combined (Figure 1b, Table 2).

### 2.2. Species Survival

**Figure 2:**
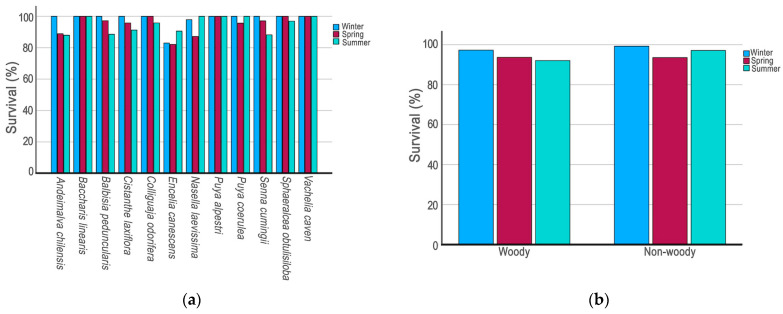
(**a**) Survival percentage during for winter, spring, and summer seasons in 12 native plant species from central Chile; (**b**) survival percentage for winter, spring, and summer seasons for woody and non-woody species combined.

The final cumulative survival rate for the 12 species combined averaged at 85.9%, and 10 showed a cumulative final survival rate of at least 85.4%. At one extreme, *B. linearis*, *P. alpestri*, and *V. caven* had a final cumulative survival rate of 100% (Figure 2a). At the other extreme, the species *A. chilensis* and *E. canescens* achieved a final cumulative survival of 75.0% and 59.6%, respectively. Even *E. canescens* achieved a statistical survival rate lower than *A. chilensis* (χ^2^ = 4.56; *p* = 0.033).

In a seasonal analysis, the 12 species showed survival rates above 80% in each season monitored (Figure 2a). Winter was the season with the highest survival rates for all species except *E. canescens*. Ten species showed 100% survival during winter (Figure 2a). The species *C. odorífera*, *P. coerulea*, and *S. obtusiloba* achieved a final survival rate of 100% in at least two seasons (Figure 2a). Four species had a final survival rate of 100% during the summer: *B. linearis*, *P. alpestri*, *P. coerulea*, and *V. caven*.

Globally, the combined non-woody species demonstrated higher survival rates during both the winter and summer months, while the combined woody species exhibited higher survival rates exclusively during the winter period (Figure 2b).

In conclusion, only the species *C. laxiflora* and *N. laevisima* showed evidence of water-sensitive survival (Table 1). In the other 10 species tested, no significant differences were found between irrigation treatments.

## 3. Discussion

Vegetation in public areas of cities has emerged as a key component of nature-based solutions in socio-ecologically stressed urban environments, although the biogeographical origin of its components has not been sufficiently emphasized in central Chile [28,29]. Several studies of Mediterranean-type climate regions around the globe suggest that native species should be preferred because they contribute more to ecosystem functioning and the ecological integrity of urban environments, and provide a greater number of ecosystem services [30,31,32,33,34]. Additionally, it could be crucial to select native species not only for their ecological features but also having evolved in particular habitats characterized by summer drought stress similar to those present in the urban area of central Chile [35]. This further inclusion could greatly expand the number of plants potentially suitable for growing in cities with Mediterranean-type climates. The present study fits within this conceptual framework.

In regions with a Mediterranean-type climate, methodologies have been designed to identify potential native species that are not used in various urban vegetation contexts. These methodologies use bibliographic information on the species ecology, distribution, and performance in their natural conditions [36]. However, it should not be forgotten that experimental evidence should also be gathered in the contexts of diverse urban habitats [37].

One set of evidence comes from assessing the condition and health of historic vegetation in different areas of the city [38]. A second set comes from managing and monitoring vegetation from the moment an urban green area is implemented, even if it was not considered an experimental design from the outset [39]. This study is closer to the second group, as it sought potential native species to be used in public areas in experimental urban contexts in a central Chile city. Although published information on native species from central Chile was used for an initial selection of potential species (see Table 3), the plants were then subjected to an experimental procedure to evaluate their performance in real urban conditions.

The results of this study showed that the irrigation gradient tested did not affect survival in 83% of the potential native species selected for use in urban areas of central Chile. The result is consistent with studies of Mediterranean species in natural conditions, where survival differences due to irrigation are not always observed, probably because these species have low water requirements [44,45,46]. In fact, the results show that overall final cumulative survival is greater than 80%, with the exception of the *E. canescens* shrub. It should be noted that the first year is a bottleneck for urban plant survival, due to the stress suffered by individuals during their transfer and transplantation, including the effects of environmental pollution [18,47]. Even, the results even showed that survival was high after summer water stress.

On the other hand, the results showed that the shoot growth of about 66% of the studied species was significantly affected by the irrigation gradient, mainly in the summer. However, only two species had their survival rates affected by the applied irrigation gradient. Furthermore, overall species growth practically stopped in winter, although survival rates were high. On the other hand, the highest shoot growth in woody and non-woody species was in the spring season. Consequently, the research shows that the species studied exhibit temporal variation in their water behavior patterns consistent with species from Mediterranean-type climates, characterized in central Chile by a winter influenced by the polar jet stream, numerous frosts with snow at higher elevations, an extended period of summer drought, and high interannual variability and temperatures [48]. However, survival was not significantly affected in both seasons. According to the literature, cold ocean currents on the west coast of Mediterranean-type climate regions moderate temperatures, allowing survival and plant growth in the late winter and early spring [49].

The results of this study showed that only the herb *N. laevissima* and the shrub *C. laxiflora* presented highly water-sensitive responses, as both growth and survival were significantly affected (Table 1). In fact, growth was negative in *C. laxiflora* and zero in *N. laevissima*, and in both species, survival was lower in the treatment with less water availability (Table 1 and Table 2). Both species concentrated their shoot growth during the spring, and the results even show that *C. laxiflora* suffered significant self-thinning during the summer. ROS (Reactive Oxygen Species) analyses, which were carried out in parallel with this study, indicate potential oxidative stress in the herb *N. laevissima* in midsummer for low irrigation treatments [50]. The environmental stress suffered by this species could explain its low abundance in natural conditions in the Metropolitan Region of central Chile [51] and its displacement by exotic annual plants that use water more efficiently [52]. On the contrary, the same ROS analysis shows the absence of oxidative stress in the *C. laxiflora* shrub in any of the treatments applied during the same period of the year, probably due to the decrease in both the transpiration surface area and water loss suffered by the species during this period of greater water stress [53]. However, *C. laxiflora* survival was negatively affected (Figure 2a), probably due to a decrease in the photosynthetic area caused by self-thinning during the summer.

The succulent herbs *P. alpestri* and *P. coerulea* stand out among the eight species of water-sensitive growth, as they have adapted their photosynthesis to avoid summer water stress [54]. Although the results showed that the species were water-sensitive growth, however survival was 100% in all treatments (Table 1). This pattern demonstrates the ability of both succulent species to colonize sites exposed to solar radiation (Table 3) and to avoid water stress in the summer [54]. Even during this season, H_2_O_2_ levels associated with water stress are normal in both succulents of this study [50]. However, during the winter, growth was zero in both species, probably indicating stress levels due to cold weather events and good resistance to the same phenomenon, as the average survival rate was close to 100%. A pattern very similar to that found in *P. alpestri* and *P. coerulea* can be seen in the shrubs *S. cumingii*, *B. linearis*, and in the tree *V. caven*. The plants of *S. cumingii* have deep roots that perform hydraulic lifting, allowing them to reach more humid soil layers and move water to dry surface layers, thus avoiding water stress during the summer [55,56]. However, we can also see that summer is the season with the highest mortality rate and the season with the highest growth rate in urban areas as well. With regard to *V. caven*, a dominant species of thorny shrubland [57] and represented in the native urban flora of central Chile [26], research shows high survival rates throughout the year, although growth has been limited under severe water restrictions. On the other hand, unlike *S. cumingii*, in the tree *V. caven* there is no differentiated use of water within the root zone, but rather constant activity to capture the water available at a depth of 100 cm [58]. The last representative of the species water-sensitive is *B. linearis*, which develops shallow roots. This shrub is suitable for colonizing barren soils and remediating soils contaminated by mining [59], making it an attractive candidate for rehabilitating urban soils with abundant fill.

Analysis of the results also detected a group of four potential species that were not water-sensitive to the treatments applied and showed high survival rates, with the exception of *E. canescens* (Table 1). This shrub from arid regions showed a very low survival rate during winter and spring (Figure 2a), which could not even be reverted with the increased water availability in the irrigation gradient applied (Table 1). Interestingly, the literature shows that *E. canescens* is a common species that is resistant to low water availability [60]. It is suggested that plants from more arid areas with similar behavior could benefit from the establishment of seedlings in shaded environments, although this hypothesis would need to be evaluated more precisely [23,61]. The ROS analysis for *E. canescens* was consistent with the results for survival and shoot growth in the water gradient (Table 1) because it showed potential oxidative stress during the summer in all treatments, and was even reinforced in the plot with low water availability. The low survival rate of *E. canescens* during winter is probably due to frost stress, which cannot be reversed and recovered from before the onset of summer water stress in plots that are completely exposed to solar radiation. In contrast, although the *S. obtusiloba* shrub also showed oxidative stress during the summer, which was intensified in the low irrigation treatment [50], its survival rate was high with growth not stopping under any water conditions. In this species, there is probably a mechanism associated with secondary metabolites such as proline, sugars, or antioxidants to prevent water stress, which should be evaluated in further studies. 

The last water-insensitive species, the shrub *B. peduncularis*, reduces its water deficit after the application of more irrigation in the summer season. The reviewed literature estimates that *B. peduncularis* responds efficiently to increases in surface water availability and is therefore dependent on rainfall and surface soil moisture [62].

Our study evaluated both plant survival and growth, although the indices used incorporate the shoot and exclude root condition and growth. Regarding survival, the monitoring of woody species did not record the regrowth of shoots that were considered dead during previous monitoring. Although regrowth was observed in perennial herbaceous, it occurred only occasionally. Furthermore, given the high survival rate of the studied species, our results suggest that the used index has not underestimated plant survival. Unfortunately, we could not incorporate root growth into the study. This would have required disruptive testing incompatible with the nature of our bio-urban shelter project and the available resources. Further studies will be needed to assess how roots compensate for losses and gains in the shoot biomass during the plant growth period to ensure the long-term sustainability of urban species used by landscape architects.

On the other hand, these survival and growth patterns may not persist in the long term. For example, there is evidence that the competitive species sown on the roof gradually gave way to stress-tolerant species [16]. However, it should be noted that our species list mainly includes long-lived woody plants that will be planted in areas with intensive urban use. These areas often prevent the spontaneous recruitment of new plants. In contrast, extensive green roofs are installed on vacant unmanaged green roofs that require low maintenance and spontaneous colonization is accepted as design criteria [16,19].

These types of studies must consider the importance of both current environmental conditions and future climate scenarios in areas undergoing urbanization in central Chile. We selected a group of species that are not only found in the central Chilean Mediterranean-type climate region, but also in more arid areas in northern Chile. Biodiversity in Mediterranean climate areas is particularly susceptible to global change [58]. Urban vegetation proposals should not only improve the adaptability of urban green infrastructure in a changing environment but also contribute to the broader goals of biodiversity conservation in a particularly vulnerable and endangered Mediterranean-type region [63,64,65]. This study is of significant practical value due to its implications for urban planning in Chile.

## 4. Materials and Methods

### 4.1. Study Site

**Figure 3:**
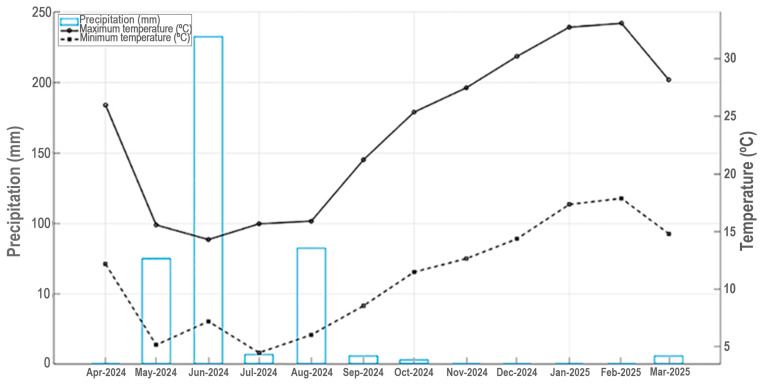
Mean monthly maximum and minimum temperature and precipitation for Universidad Central de Chile, Santiago, for April 2024 to March 2025.

Santiago (33° S; 70° W; 550 m.a.s.l.), the Chilean capital, has a Mediterranean climate type, characterized by mild wet winters and warm dry summers (Figure 3) [45,66]. The Santiago metropolitan area currently has about 6.3 million people, with a population density of roughly 8497 inhabitants per Km^2^ [67]. Since the late 20th and early 21st centuries, urban growth has also spread to surrounding areas, mostly consisting of agricultural lands and smaller remnants of semi-natural vegetation [68,69,70]. Urban vegetation of Santiago is dominated by exotic flora, which represents more than 80% of the total urban flora [26]. Water scarcity in Santiago metropolitan area has been increasing in recent years, associated with a sustained decrease in rainfall and the effects of climate change [24]. We chose Santiago as a study site in central Chile due to the Bio-Urban Shelter project designed for public area in Santiago [71].

### 4.2. Species Selection

**Figure 4:**
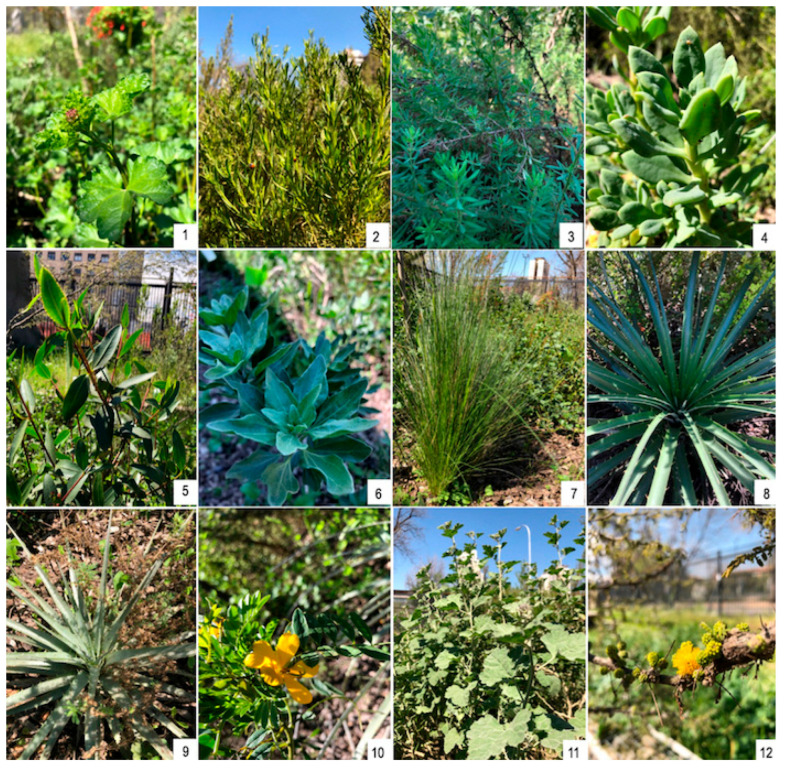
The twelve native plant species from central Chile that were selected for the experimental study. 1. *Andeimalva chilensis*, 2. *Baccharis linearis*, 3. *Balbisia peduncularis*, 4. *Cistanthe laxiflora*, 5. *Colliguaja odorífera*, 6. *Encelia canescens*, 7. *Nasella laevissima*, 8. *Puya alpestri,* 9. *Puya coerulea,* 10. *Senna cumingii,* 11. *Sphaeralcea obtusiloba* and 12. *Vachellia caven*.

We used the landscape value method to select 12 potential native plant species from central Chile for use in urban vegetation in the same region (Figure 4) [36]. The method consists of four selection criteria: (a) environmental criteria, which are native species adapted to the environment where they will be used; (b) aesthetic criteria, morphological features that have aesthetic value for the landscape use of the species; (c) cultural criteria, which refer to the meanings and values of the use of the flora; and (d) management criteria, which is the ability of the species to respond to management and conditions in public area. Consideration was given to ensuring that the selected species were available in nurseries in the region as well, due to the potential demand for Bio-Urban Shelter in public areas of Santiago by local governments.

Consequently, the selected native plants are represented by trees, shrubs, and perennial herbs (Table 3), which are mostly common species of the thorny shrublands of central Chile and dominant species of a potential community within the urban perimeter of Santiago [72].

### 4.3. Experimental Design

The experimental plots are located in the historic center of the city of Santiago, Chile. To consider the environmental variation in Santiago, two university campus in the historic center were selected. Two experimental plots were set up on the campus of the Universidad Tecnológica Metropolitana (UTEM, 33.451071° S, 70.656622° W) and two similar plots at the Universidad Central de Chile (UCEN; 33.451360° S, 70.653433° W).

**Figure 5:**
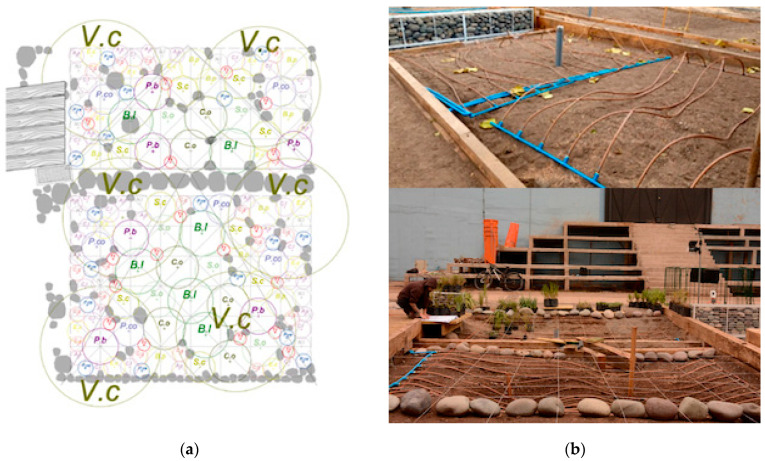
(**a**) Planting plan for the four experimental plots. Two plots were located Universidad Central de Chile (UCEN) and another two Universidad Tecnológica Metropolitana (UTEM), in the historic center of the city of Santiago, central Chile. Vc = *Vachelia caven*, cl = *Cistanthe laxiflora,* nl = *Nasella laevissima*, Ac = *Andeimalva chilensis*, Co = *Colliguaja odorífera*, Pa *= Puya alpestri*, Pco = *Puya coerulea*, *Senna cumingii*, Bl = *Baccharis linearis*, Bp = *Balbisia peduncularis*, Ec = *Encelia canescens*, So = *Sphaeralcea obtusiloba*; (**b**) long-lasting, low-yield automated underground drip system with a pipe with a built-in emitter and Copper Shield technology.

Each plot had an area of 20 m^2^. The soil in the plots was homogenized with a structure suitable for plant growth. Consequently, 60% of the existing soil in the top 150 cm of the plots was cleaned and loosened for recovery. The remaining 50% of the planting substrate consisted of 20% coarse sand, 15% gravel, and 5% compost, which increased the organic matter in the soil. A 5 cm layer of organic mulch was also added to the surface to prevent weed growth and excessive water loss from the soil. On average, soil pH = 7.8, nitrogen = 32 mg/kg, phosphorus = 67 mg/kg and potassium = 374 mg/kg. The 12 native species selected from central Chile, including woody and herbaceous perennials, were planted uniformly within each plot to reduce competition, cover spatial variation and avoid pseudo replicates (Figure 5a). The replicates per species are reported in Table 1. The spatial location and arrangement of the species was the same in the four experimental plots and responded to landscape and monitoring criteria, as the experiment is part of a project on bio-urban shelters in public area in Santiago city.

**Figure 6:**
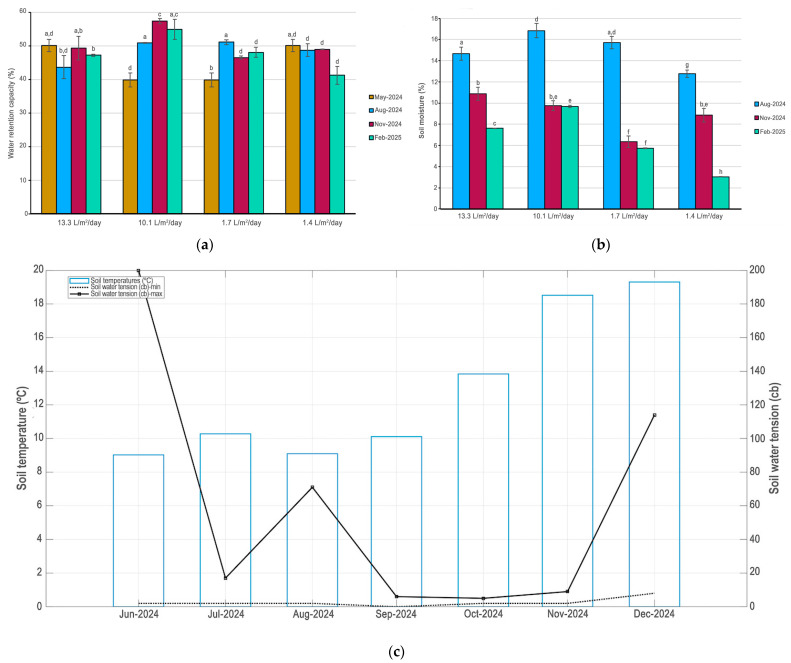
(**a**) Water retention capacity of the soil. Bars represent ±1 E.D. Different letters indicate statistically significant differences among WRC assessed according to Bonferroni test (*p* ≤ 0.05); (**b**) soil moisture. Bars represent ±1 E.D. Different letters indicate statistically significant differences among soil moisture assessed according to Bonferroni test (*p* ≤ 0.05); (**c**) Mean monthly maximum and minimum soil water tension and temperature for Universidad Central de Chile campus, Santiago, for June 2024 to December 2024.

Juvenile plants under two years old were obtained from certified nurseries and, after being purchased, were immediately planted in the plots. The soil was saturated with water to prevent stress and mortality when planting. The plants at UTEM were planted between 29 and 31 May 2024. At UCEN, they were planted between 18 and 20 June 2024. The experiment lasted from 29 June 2024 (the initial time) to 3 March 2025 (the final time). The irrigation system installed was a long-lasting, low-yield automated underground drip system with a pipe with a built-in emitter and Copper Shield technology (Figure 5b), which protects the dripper from root intrusion [73]. The hose has a diameter of 16 mm and a flow rate of 2.3 L/h (ESP, Rain Bird, Azusa, CA, USA). Irrigation is controlled automatically (X-Core, 601I, 230 VCA, Hunter, New York, NY, USA). The system was fed by the drinking water system. In each plot, the irrigation lines were buried at a depth of 30 cm and spaced 30.5 cm apart. In the center of the plots were installed soil humid and temperature sensors (Vantage Pro2 and Environmonitor, Sku 6440, Davis Instruments, Hayward, CA, USA). The soil water conditions and temperature for plots were studied (Figure 6a–c). Records demonstrated that water gradient provided by irrigation remained in the soil during the summer or February 2025 (Figure 6b).

Each campus had one plot with high water availability for plants and another with low water availability with the aim of generating a water gradient in the experimental plots (Table 4). The 2 plots located at UCEN had an average daily irrigation regime of 13.3 L/m^2^/day and 1.4 L/m^2^/day. Meanwhile, the 2 plots located at UTEM were irrigated daily during the irrigation season with an average of 10.1 L/m^2^/day and 1.7 L/m^2^/day. Irrigation was maintained throughout the summer months on all four experimental plots, even after the experiment ended. Therefore, the plots were not watered between 29 June and 23 November 2024. The result was that each plot was subject to a different water regime during the irrigation period. Finally, the water gradient applied represents the recommended irrigation for public green areas in the Santiago Metropolitan Region at the upper end, and conditions considered to be water stress for urban plants at the lower end [74].

### 4.4. Statistical Design

To evaluate survival in woody and herbaceous perennial species, plant shoots were visually monitored at the end of winter (13 September 2024), the end of spring (17 December 2024), and the end of summer (3 March 2024). If the plant shoot was completely dry, the individual was considered dead, considering that it could subsequently regrow at the base of the shoot and its condition could change to alive. To determine the effect of water treatment on species survival, Kaplan–Meier survival analyses were performed. Similarly, to determine the effect of water treatment on the survival of woody versus non-woody species in combination. This analysis is based on estimating the conditional probabilities of survival at each monitoring event or season (winter, spring, and summer) and considering the limit of the product of those probabilities to estimate the survival rate at each event.

The growth of each individual will be estimated using the following Growth Index:

GI = (A + B + C)/3

A = plant height; B = canopy length; C = canopy width.

The growth per individual for each season of the year was estimated by:

GI_t1_ − GI_t0_

t1 = end of the current year season,

t0 = immediately preceding year season.

The GI of each individual was monitored at the end of winter (14 September 2024), the end of spring (18 December 2024), and the end of summer (4 March 2025).

The GI was analyzed using a GLM of repeated measures. This analysis determines the effect of the four irrigation treatments on the GI by performing inter-subject tests. Bonferroni was used for comparisons between pairs of treatments. The data were transformed to log_10_ if they did not meet normality and homoscedasticity. The effect of the year season and its interaction with the treatments was determined using intra-subject effect tests. When the sphericity condition was not met according to Mauchly’s test, the Greenhouse-Geisser correction or, failing that, the Huynh-Feldt correction was performed in the intra-subject effect tests. Similarly, water retention capacity and moisture of the soil was analyzed using a GLM. A significance level of *p* < 0.05 and confidence interval = 95% were considered in the analyses. Statistical analyses were performed using the IBM SPSS Statistics for PC, version 30.0 (IBM SPSS, Armonk, NY, USA: IBM Corp. 2024).

For the purposes of this study: we defined a species as being highly water-sensitive if significant differences in plant growth and survival were observed between the different water treatments applied during the trial. A species is water-sensitive if significant differences in plant growth or survival is observed between the different water treatments applied during the trial. Finally, a species is considered non-sensitive if there are no significant differences in plant growth and survival between water treatments.

## 5. Conclusions

Survival results show that almost 80% of the native central Chileans species selected for this study were not affected by the water treatments applied and even reached high percentages in the first year of establishment, which is considered a survival bottleneck for urban plants. However, when water resources were severely limited, shoot growth can be slowed significantly, because they are water-sensitive species. The study also showed that the main growing season for both woody and non-woody species is spring. Growth is then slowed during the summer, with even biomass being lost. Finally, growth is stopped during the winter. Nevertheless, the survival rates may be high in each season.

In concluding, the evidence obtained in this study suggests that native species in central Chile can achieve high survival rates with limited irrigation. This highlights their potential for use in the sustainable urban green infrastructure of cities located in Mediterranean-type climates, which are expected to experience low rainfall due to climate change. Using native plants in urban green areas helps create more resilient plant communities and generate a broader array of ecological benefits.

Nevertheless, certain limitations persist. Resources should be focused on increasing the number of experimental plots and considering a randomized design from the outset. This calls for additional investigation into the potential use of native plants in a landscape that varies in both space and time.

## Figures and Tables

**Table 1:** Shoot growth index (±1 E.D.) and survival rate (final %) for each irrigation treatment applied to the 12 species of native plants from central Chile tested in the experimental plots. N = replicates per experimental plot. Responses: Highly sensitive = Growth and survival with significant *p*-Value; Sensitive = Growth or survival with significant *p*-Value. Non-sensitive = Growth and survival with non-significant *p*-Value. Different letters indicate significant differences between treatments (*p* < 0.05) Note: * *p* ≤ 0.05; ** *p* ≤ 0.01; *** *p* ≤ 0.001; n.s. = *p* > 0.05.

Species	N	Dependent Variable	13.3 L/m^2^/Day	10.1 L/m^2^/Day	1.7 L/m^2^/Day	1.4 L/m^2^/Day	*p*-Value	Responses
*Cistanthe laxiflora*	12	Growth	2.0 (1.4) ^a,b^	2.8 (1.4) ^a^	0.4 (1.4) ^a,b^	−3.2 (1.4) ^b^	*	Highly sensitive
		Survival	83.3 ^a,b^	100 ^a^	91.7 ^a,b^	75.0 ^b^	*	
*Nasella laevissima*	12	Growth	7.4 (1.2) ^a^	10.5 (1.2) ^a^	7.9 (1.2) ^a^	0.9 (1.2) ^b^	***	Highly sensitive
		Survival	83.3 ^a,b^	91.7 ^a,b^	100 ^a^	66.7 ^b^	*	
*Andeimalva chilensis*	18	Growth	22.3 (3.3) ^a^	17.7 (3.3) ^a^	5.6 (3.3) ^b^	11.6 (3.3) ^a,b^	**	Sensitive
		Survival	83.3	72.2	61.1	83.3	n.s.	
*Colliguaja odorifera*	6	Growth	10.7 (2.1) ^a^	5.0 (2.1) ^a,b^	1.4 (2.1) ^b^	3.5 (2.1) ^a,b^	*	Sensitive
		Survival	100	100	83,3	100	n.s.	
*Puya alpestri*	6	Growth	5.6 (1.1) ^a^	3.6 (1.1) ^a,b^	1.2 (1.1) ^b^	1.3 (1.1) ^b^	*	Sensitive
		Survival	100	100	100	100	n.s.	
*Puya coerulea*	6	Growth	3.8 (0.8) ^a^	3.6 (0.8) ^a^	0.02 (0.9) ^b^	0.02 (0.9) ^b^	**	Sensitive
		Survival	100	100	100	80	n.s.	
*Senna cumingii*	9	Growth	11.0 (2.2) ^a^	4.1 (2.3) ^a,b^	−0.4 (2.2) ^b^	4.7 (2.2) ^a,b^	**	Sensitive
		Survival	88.9	75,0	77,8	100	n.s.	
*Vachelia caven*	6	Growth	16.7 (2.7) ^a,b^	21.6 (2.7) ^a^	6.2 (2.7) ^b^	7.3 (2.7) ^b^	***	Sensitive
		Survival	100	100	100	100	n.s.	
*Baccharis linearis*	6	Growth	15.3 (2.1)	19.5 (2.1)	19.5 (2.1)	12.4 (2.1)	n.s.	Non-sensitive
		Survival	100	100	100	100	n.s.	
*Balbisia peduncularis*	9	Growth	8.1 (2.7)	3.1 (2.7)	7.6 (2.7)	6.2 (2.7)	n.s.	Non-sensitive
		Survival	77.8	66.7	100	100	n.s.	
*Encelia canescens*	12	Growth	0.9 (2.9)	4.1 (2.9)	5.4 (2.9)	2.9 (3.1)	n.s.	Non-sensitive
		Survival	41.7	58.3	75.0	63.3	n.s.	
*Sphaeralcea obtusiloba*	8	Growth	13.9 (3.0)	16.0 (3.5)	7.9 (3.0)	11.2 (3.0)	n.s.	Non-sensitive
		Survival	100	100	87.5	100	n.s.	

**Table 2:** Generalized linear model of repeated measures results showing the effect Intra-subject, contrast Intra-subject and of the four irrigation treatments on the growth index by performing Inter-subject tests. N = replicates per experimental plot. TIME = Season. TREAT = Treatment. Note: * *p* ≤ 0.05; ** *p* ≤ 0.01; *** *p* ≤ 0.001; n.s. = *p* > 0.05.

Species	N	Effect Intra-Subject				Contrast Intra-Subject				Effect Inter-Subject	
		TIME		TIME × TREAT		TIME		TIME × TREAT		TREAT	
		F-Value	*p*-Value	F-Value	*p*-Value	F-Value	*p*-Value	F-Value	*p*-Value	F-Value	*p*-Value
*Andeimalva chilensis*	18	41.43	***	4.00	**	79.58	***	5.37	**	4.78	**
*Baccharis linearis*	6	36.96	***	2.61	*	63.80	***	2.67	n.s.	2.68	n.s.
*Balbisia peduncularis*	9	7.71	**	1.62	n.s.	17.03	***	1.71	n.s.	0.70	n.s.
*Cistanthe laxiflora*	12	10.09	**	0.29	n.s.	10.24	**	0.11	n.s.	3.61	*
*Colliguaja odorifera*	6	4.34	*	3.21	*	6.04	*	4.88	*	3.80	*
*Encelia canescens*	12	12.25	***	0.97	n.s.	20.26	***	0.57	n.s.	0.74	n.s.
*Nasella laevissima*	12	30.19	***	3.53	**	33.20	***	0.34	n.s.	11.33	***
*Puya alpestri*	6	13.82	***	5.74	***	18.74	***	5.48	**	3.55	*
*Puya coerulea*	6	7.71	**	2.60	n.s.	18.19	***	6.30	**	6.10	**
*Senna cumingii*	9	7.59	**	2.33	n.s.	10.35	**	2.37	n.s.	4.71	**
*Sphaeralcea obtusiloba*	8	19.65	***	3.38	**	27.26	***	5.06	**	1.2	n.s.
*Vachelia caven*	6	20.31	***	4.59	**	47.77	***	6.92	**	7.59	**
Non-woody	35	21.03	***	1.45	n.s.	34.11	***	0.09	n.s.	8.63	***
Woody	75	63.15	***	8.27	***	46.97	***	7.44	***	5.73	***

**Table 3:** Plant species from central Chile selected for the study, indicating family affiliation, life form, and environmental, aesthetic, cultural, and management criteria considered in the potential landscape value of the species for use in urban vegetation in central Chile. Information obtained in [40,41,42,43].

Species	Family	Life Form	Criteria			
			Environmental	Aesthetic	Cultural	Management
*Cistanthe* *laxiflora*	Montiaceae	Perennial herb	Endemic. Tolerant of saline soils, poor in nutrients, and low moisture. Flowers attract native pollinators	Pink fuchsia flowers in spring and summer	No information	Plant in full sun in association with many species
*Nasella* *laevissima*	Poaceae	Perennial herb	Endemic. Tolerant in low-moisture soils and poor in nutrients and eroded.	Species with diverse shapes, colors, and subject to the wind	No information	Plant in full sun. Fast growth rate
*Andeimalva* *chilensis*	Malvaceae	Shrub	Endemic. Tolerant of saline soils, poor in nutrients, and low moisture. Attracts a diversity of floral fauna.	Extensive pink coloration and attractive appearance of flowering	No information	Plant in full sun, tolerates pruning, and resistant to pests and diseases
*Colliguaja* *odorifera*	Euphorbiaceae	Shrub	Endemic. Tolerant in low-moisture soils and poor in nutrients.	Edges of leaves and flowers are reddish in color. Ballistic seed dispersal	Medicinal. Insecticide. Dyeing. Handcrafted	Plant in full sun. Fast growth rate, resists pruning
*Puya alpestri*	Bromeliaceae	Perennial herb	Endemic. Tolerant in low-moisture soils and poor in nutrients. Attracts hummingbirds and butterflies	Attractive colors, textures, structure, and shapes of the plant. Flowers in spring and fruits in summer	Edible	Plant in full sun and resists pruning
*Puya coerulea*	Bromeliaceae	Perennial herb	Endemic. Tolerant in low-moisture soils and poor in nutrients. Attracts hummingbirds and butterflies	Attractive colors, textures, structure, and shapes of the plant. Flowers in spring and fruits in summer	No information	Plant in full sun and resists pruning
*Senna cumingii*	Fabaceae	Shrub	Endemic. Tolerant of saline soils, poor in nutrients, low moisture and fixes nitrogen	Golden yellow flowers in spring and summer	No information	Plant in full sun
*Vachelia caven*	Fabaceae	Tree	Endemic. Tolerant of saline soils, poor in nutrients, low moisture and fixes nitrogen. Attracts birds	A variety of colors throughout the year	Edible. Medicinal. Fuel. Handcrafted. Dye	Plant in full sun. Fast growth rate
*Baccharis* *linearis*	Asteraceae	Shrub	Native. Tolerant in low-moisture soils and eroded. Attracts pollinators	Attractive colors, textures, structure, and shapes of the plant	Medicinal	Plant in full sun. Fast growth rate, tolerates pruning, and resistant to pests and diseases
*Balbisia* *peduncularis*	Vivianiaceae	Shrub	Native. Tolerant of saline soils, poor in nutrients, and low moisture.	Attractive flowers in spring and summer.	No information.	Plant in full sun.
*Encelia* *canescens*	Asteraceae	Shrub	Native. Tolerant in low-moisture soils and poor in nutrients. Attracts native pollinating bees	Yellow flowers in spring and fall	No information	Plant in full sun. Fast growth rate
*Sphaeralcea* *obtusiloba*	Malvaceae	Shrub	Endemic. Tolerant in low-moisture soils and poor in nutrients. Attracts native pollinating bees	Lilac flowers in spring and fall. Attractive colors, textures, and shapes of the foliage	Medicinal	Plant in full sun. Fast growth rate and resistant to pests

**Table 4:** Initial and final irrigation dates for the experimental plots set up on the UCEN and UTEM campus. The plots are labeled according to the average daily amount of irrigation applied throughout the season. Irrigation began on 23 November 2024, when the soil reached 100 cb for the first time during the experiment. On 2 December 2024, the soil reached 200 cb for the first time. UCEN = Universidad Central de Chile; UTEM = Universidad Tecnológica Metropolitana.

	UCEN		UTEM	
Average irrigation (L/m^2^/day)	13.3	1.4	10.1	1.7
Initial date	23 November 2024	2 December 2024	2 December 2024	2 December 2024
Final date	19 March 2025			

## Data Availability

The data presented in this study are available on request from the corresponding author J.F.

## References

[1] Liu X., Li C., Zhao X., Zhu T. (2024). Arid Urban Green Areas Reimagined: Transforming Landscapes with Native Plants for a Sustainable Future in Aksu, Northwest China. Sustainability.

[2] McPherson E.G., Platt R.H., Rowntree R.A., Muick P.C. (1994). Cooling urban heat islands with sustainable landscapes. The Ecological City: Preserving and Restoring Urban Biodiversity.

[3] Richter M., Heinemann K., Meiser N., Dickhaut W. (2024). Trees in sponge cities—A systematic review of trees as a component of blue-green infrastructure, vegetation engineering principles, and Stormwater management. Water.

[4] Catalano C., Laudicina V.A., Badalucco L., Guarino R. (2018). Some European green roof norms and guidelines through the lens of biodiversity: Do ecoregions and plant traits also matter?. Ecol. Eng..

[5] Rasoolzadeh R., Mobarghaee N., Esmaeilzadeh H., Rashidi Y., Marcu M.V., Sadeghi S.M.M. (2024). Carbon sequestration and storage of urban trees in a polluted semiarid city. Forests.

[6] Varela-Stasinopoulou D.S., Nektarios P.A., Ntoulas N., Trigas P., Roukounakis G.I. (2023). Sustainable Growth of Medicinal and Aromatic Mediterranean Plants Growing as Communities in Shallow Substrate Urban Green Roof Systems. Sustainability.

[7] Burch W.R., Grove J.M. (1993). People, trees and participation on the urban frontier. Unasylva.

[8] Dwyer J.F., McPherson E.G., Schroeder H.W., Rowntree R.A. (1992). Assessing the benefits and costs of the urban forest. J. Arboric..

[9] Simpson J.R., McPherson E.G. (2007). San Francisco Bay Area State of the Urban Forest Final Report.

[10] Faber Taylor A., Kuo F.E. (2011). Could exposure to everyday green spaces help treat ADHD? Evidence from children’s play settings. Appl. Psychol. Health Well-Being.

[11] Bele A., Chakradeo U. (2021). Public perception of biodiversity: A literature review of its role in urban green spaces. J. Land. Ecol..

[12] Lohr V.I., Pearson-Mims C.H., Tarnai J., Dillman D.A. (2004). How urban resident rate and rank the benefits and problems associated with trees in cities. Arboric. Urban For..

[13] Irarrázaval F. (2012). El imaginario” verde” y el verde urbano como instrumento de consumo inmobiliario: Configurando las condiciones ambientales del área Metropolitana de Santiago. Rev. INVI.

[14] Uribe S.V., Villaseñor N.R. (2024). Inequities in urban tree care based on socioeconomic status. Urban For. Urban Green..

[15] Kornienko V., Reuckaya V., Shkirenko A., Meskhi B., Olshevskaya A., Odabashyan M., Shevchenko V., Teplyakova S. (2025). Silvicultural and Ecological Characteristics of *Populus bolleana* Lauche as a Key Introduced Species in the Urban Dendroflora of Industrial Cities. Plants.

[16] Catalano C., Marcenò C., Laudicina V.A., Guarino R. (2016). Thirty years unmanaged green roofs: Ecological research and design implications. Landsc. Urban Plan..

[17] Allen K.S., Harper R.W., Bayer A., Brazee N.J. (2017). A review of nursery production systems and their influence on urban tree survival. Urban For. Urban Green..

[18] de la Fuente L.M., Ovalle J.F., Arellano E.C., Ginocchio R. (2018). Does woody species with contrasting root architecture require different container size in nursery?. Madera Bosques.

[19] Aloisio J.M., Palmer M.I., Tuininga A.R., Lewis J.D. (2020). Introduced and native plant species composition of vacant unmanaged green roofs in New York City. Urban Ecosyst..

[20] Esperon-Rodriguez M., Rymer P.D., Power S.A., Barton D.N., Cariñanos P., Dobbs C., Eleuterio A.A., Escobedo F.J., Hauer R., Hermy M. (2022). Assessing climate risk to support urban forests in a changing climate. Plants People Planet.

[21] Kim Y.J., Yoo G. (2021). Suggested key variables for assessment of soil quality in urban roadside tree systems. J. Soils Sediments.

[22] Vico G., Thompson S.E., Manzoni S., Molini A., Albertson J.D., Almeida-Cortez J.S., Fay P.A., Feng X., Guswa A.J., Liu H. (2015). Climatic, ecophysiological, and phenological control son plant ecohydrological strategies in seasonally dry ecosystems. Ecohydrology.

[23] Yáñez M.A., Espinoza S.E., Magni C.R., Martínez-Herrera E. (2024). Early Growth and physiological acclimation to shade and water restriction of seven sclerophyllous species of the Mediterranean forests of central Chile. Plants.

[24] Rodríguez C., Serrano J., Sánchez R., Leiva E. (2024). The Hydrosocial Cycle and the Inequalities in Access to Water in Rural Areas of Metropolitan Region of Santiago, Chile. Water.

[25] Fuentes I., Fuster R., Avilés D., Vervoort W. (2021). Water scarcity in central Chile: The effect of climate and land cover changes on hydrologic resources. Hydrol. Sci. J..

[26] Figueroa J.A., Teillier S., Guerrero-Leiva N., Ray-Bobadilla C., Rivano S., Saavedra D., Castro S.A. (2016). Flora vascular en el espacio público de Santiago, Chile. Gayana Bot..

[27] Ekren E., Çorbacı Ö.L., Kordon S. (2024). Evaluation of plants based on ecological tolerance criteria: A case study of urban open green spaces in Rize, Türkiye. Turk. J. For. Sci..

[28] Figueroa J.A., Castro S.A., Reyes M., Teillier S. (2018). Urban park area and age determine the richness of native and exotic plants in parks of a Latin American city: Santiago as a case study. Urban Ecosyst..

[29] Guevara B.R., Uribe S.V., de la Maza C.L., Villaseñor N.R. (2024). Socioeconomic disparities in urban forest diversity and structure in green areas of Santiago de Chile. Plants.

[30] Blasi C., Biondi E., Izco J. (2011). 100 years of plant sociology: A celebration. Plant Biosyst.-Int. J. Deal. All Asp. Plant Biol..

[31] Säumel I., Weber F., Kowarik I. (2016). Toward livable and healthy urban streets: Roadside vegetation provides ecosystem services where people live and move. Environ. Sci. Policy.

[32] Conway T.M., Almas A.D., Coore D. (2019). Ecosystem services, ecological integrity, and native species planting: How to balance these ideas in urban forest management?. Urban For. Urban Green..

[33] Arcos-LeBert G., Aravena-Hidalgo T., Figueroa J.A., Jaksic F.M., Castro S.A. (2021). Native trees provide more benefits than exotic trees when ecosystem services are weighted in Santiago, Chile. Trees.

[34] D’Amato L., Bartoli F., Savo V., Caneva G. (2025). Promoting native biodiversity: An evaluation of multifactorial and bioclimatic selection criteria for street trees in Italian cities. Urban For. Urban Green..

[35] Rundel P.W., Rundel P.W., Montenegro G., Jaksic F.M. (1998). Landscape disturbance in Mediterranean-type ecosystems: An overview. Landscape Disturbance and Biodiversity in Mediterranean-Type Ecosystems.

[36] Fernández F., Delaunoy J., Chiang L., Reyes M., Figueroa J.A., Lazzoni I. (2025). Metodología de valor paisajístico: Selección de plantas nativas para la infraestructura verde pública de la Región Metropolitana. Biodiversidad Urbana en Chile: Estado del Arte y los Desafíos Futuros.

[37] Grimm N.B., Pickett S.T., Hale R.L., Cadenasso M.L. (2017). Does the ecological concept of disturbance have utility in urban social–ecological–technological systems?. Ecosyst. Health Sustain..

[38] Degerickx J., Roberts D.A., McFadden J.P., Hermy M., Somers B. (2018). Urban tree health assessment using airborne hyperspectral and LiDAR imagery. Int. J. Appl. Earth Obs. Geoinf..

[39] Ward E.B., Doroski D.A., Felson A.J., Hallett R.A., Oldfield E.E., Kuebbing S.E., Bradford M.A. (2021). Positive long-term impacts of restoration on soils in an experimental urban forest. Ecol. Appl..

[40] Agrupación de Especies Nativas Según Condiciones Agroecológicas Aptas para su Cultivo. https://www.pumahuida.cl/wp-content/uploads/2022/03/AGRUPACIÓN-DE-ESPECIES-NATIVAS-SEGÚN-CONDICIONES-AGROECOLÓGICAS-APTAS-PARA-SU-CULTIVO-M.-MUSALEM_VI-CONGRESO-DE-FLORA-NATIVA-2019_opt.pdf.

[41] Listas de Especies Nativas para Distintas Situaciones de Paisaje. https://www.pumahuida.cl/informacion-tecnica/.

[42] Rodríguez R., Marticorena C., Alarcón D., Baeza C., Cavieres L., Finot V.L., Fuentes N., Kiessling A., Mihoc M., Pauchard A. (2018). Catálogo de las plantas vasculares de Chile. Gayana Bot..

[43] Hoffmann J.A. (2012). Flora Silvestre de Chile. Zona Central. Una Guía para la Identificación de las Especies Vegetales Más Frecuentes.

[44] Armesto J.J., Pickett S.T.A. (1985). Experiments on disturbance in old-field plant communities: Impact on species richness and abundance. Ecology.

[45] Becerra P.I., Cruz G., Ríos S., Castelli G. (2013). Importance of irrigation and plant size in the establishment success of different native species in a degraded ecosystem of central Chile. Bosque.

[46] Becerra P.I., Arellano E.C., Vilagrosa A., Hernández G., Figueroa C. (2024). The provision of water and shade but not soil amendments in degraded habitats increases the seedling survival of woody species in restoration processes of the Chilean sclerophyllous forest. Trees.

[47] Razzaghmanesh M., Beecham S., Kazemi F. (2014). The growth and survival of plants in urban green roofs in a dry climate. Sci. Total Environ..

[48] Armesto J.J., Arroyo M.T.K., Hinojosa F.L., Veblen T.T., Young K.R., Orme A.R. (2007). The Mediterranean environment of central PM Chile. The Physical Geography of South America.

[49] Rundel P.W., Arroyo M.T., Cowling R.M., Keeley J.E., Lamont B.B., Vargas P. (2016). Mediterranean biomes: Evolution of their vegetation, floras, and climate. Annu. Rev. Ecol. Evol. Syst..

[50] Chandía-Jaure R., Cataldo-Cunich A., Fernández-Cano F., Figueroa J.A., Godoy-Donoso D. (2025). Refugios biourbanos: Guía para evaluar la efectividad de soluciones basadas en la naturaleza para el diseño urbano sensible al agua, como estrategia de adaptación al cambio climático.

[51] Teillier S., Macaya-Berti J., García N., Marticorena A., Rojas G., Niemeyer H.M. (2022). Flora de la Región Metropolitana de Santiago. Guía para la Identificación de las Especies.

[52] Everard K., Seabloom E.W., Harpole W.S., De Mazancourt C. (2010). Plant water use affects competition for nitrogen: Why drought favors invasive species in California. Am. Nat..

[53] Burlett R., Trueba S., Bouteiller X.P., Forget G., Torres-Ruiz J.M., Martin-StPaul N.K., Parise C., Cochard H., Delzon S. (2025). Minimum leaf conductance during drought: Unravelling its variability and impact on plant survival. New Phytol..

[54] Ortuño M.A., Machtig A.E., Chacón M.A., Cuzmar J., Fontúrbel F.E. (2019). Spatial distribution of *Puya coerulea* Lindl. in response to abiotic factors and accompanying species in the Río Clarillo National Reserve. Gayana Bot..

[55] Prieto I., Martínez-Tillería K., Martínez-Manchego L., Montecinos S., Pugnaire F.I., Squeo F.A. (2010). Hydraulic lift through transpiration suppression in shrubs from two arid ecosystems: Patterns and control mechanisms. Oecologia.

[56] León M.F., Squeo F.A., Gutiérrez J.R., Holmgren M. (2011). Rapid root extension during water pulses enhances establishment of shrub seedlings in the Atacama Desert. J. Veg. Sci..

[57] Gerstmann C., Miranda M., Condal A. (2010). Description of space-time variability of the potential productivity of *Acacia caven* espinales based on MODIS images and the Enhanced Vegetation Index (EVI). Int. J. Agric. Nat. Res..

[58] Sepúlveda M., Bown H.E., Fernandez B. (2018). Stomatal conductance responses of *Acacia caven* to seasonal patterns of water availability at different soil depths in a Mediterranean savanna. Water.

[59] Ginocchio R., de la Fuente L.M., Orrego F., Díaz M.J., Báez J., Ovalle J.F. (2021). A novel fast-vegetative propagation technique of the pioneer shrub *Baccharis linearis* on mine tailings by adding compost. Int. J. Phytoremediat..

[60] Martínez-Tillería K., Loayza A.P., Sandquist D.R., Squeo F.A., Ward D. (2012). No evidence of a trade-off between drought and shade tolerance in seedlings of six coastal desert shrub species in north-central Chile. J. Veg. Sci..

[61] Carvajal D.E., Loayza A.P., López R.P., Toro P.J., Squeo F.A. (2014). Growth and early seedling survival of four Atacama Desert shrub species under experimental light and water availability regimes. Rev. Chil. Hist. Nat..

[62] Torres R., Squeo F.A., Jorquera C., Aguirre E., Ehleringer J.R. (2002). Evaluación de la capacidad estacional de utilizar eventos de precipitación en tres especies de arbustos nativos de Chile con distintos sistemas radiculares. Rev. Chil. Hist. Nat..

[63] Newbold T., Oppenheimer P., Etard A., Williams J.J. (2020). Tropical and Mediterranean biodiversity is disproportionately sensitive to land-use and climate change. Nat. Ecol. Evol..

[64] Caneva G., Kumbaric A., Savo V., Casalini R. (2015). Ecological approach in selecting extensive green roof plants: A data-set of Mediterranean plants. Plant Biosyst..

[65] Bambach N., Meza F.J., Gilabert H., Miranda M. (2013). Impacts of climate change on the distribution of species and communities in the Chilean Mediterranean ecosystem. Reg. Environ. Change.

[66] McPhee J., Cortés G., Rojas M., García L., Descalzi A., Vargas L., Krellenberg K., Hansjürgens B. (2014). Downscaling climate changes for Santiago: What effects can be expected?. Climate Adaptation Santiago.

[67] (2017). Densidad de Población y Vivienda. Censo. https://storymaps.arcgis.com/stories/fc03ad1481f44b6299b81c22c91497fe.

[68] De Mattos C., Hidalgo R., Arenas F., Coll J.L. (2003). Globalización y transformación metropolitana en el caso de Santiago. Los Nuevos Modos de Gestión de la Metropolización.

[69] Romero H., Vásquez A. (2005). Evaluación ambiental del proceso de urbanización de las cuencas del piedemonte andino de Santiago de Chile. Eur. Secur..

[70] Romero H., Molina M., Moscoso C., Sarricolea P., Smith P., Vásquez A., de Mattos C., Hidalgo R. (2007). Caracterización de los cambios de usos y coberturas de suelo causados por la expansión urbana de Santiago, análisis de sus factores explicativos e inferencias ambientales. Movilidad Espacial y Reconfiguración Metropolitana.

[71] Chandia-Jaure R., Cataldo-Cunich A., Bustamante-Oleart C., Fernandez Cano F., Figueroa J.A., Villagrán-Escobar M. Bio-Urban Shelters. Neighborhood-scale intervention model for water sensitive urban design. Proceedings of the 14° Encuentro Diseño Urbano, Readu.

[72] Luebert F., Pliscoff P. (2017). Sinopsis Bioclimática y Vegetacional de Chile.

[73] Ivelic-Sáez J., Reckmann O., López R., Uribe H., Valenzuela J., Ibarra D. (2021). Bases para el riego en Magallanes. Boletín INIA.

[74] Fernández F., Chiang L., Figueroa J.A. (2025). Guía de Recomendaciones para Jardines Eficientes en el Espacio Público en la Región Metropolitana.

